# COVID-19 Outbreak Perception in Italian Dentists

**DOI:** 10.3390/ijerph17113867

**Published:** 2020-05-29

**Authors:** Alberto De Stefani, Giovanni Bruno, Sabrina Mutinelli, Antonio Gracco

**Affiliations:** Department of Neuroscience, Section of Dentistry, University of Padua, 35100 Padua, Italy; alberto.de.stefani@hotmail.it (A.D.S.); sabrinamutinelli109@gmail.com (S.M.); antonio.gracco@unipd.it (A.G.)

**Keywords:** COVID-19, SARS-CoV-2, dentistry, questionnaire, infection control

## Abstract

The aim of this study is an evaluation of the Italian dentists’ knowledge regarding COVID-19 and their perception of the risks associated with COVID-19, their attitude in resuming their activities, and how they judge the institutional intervention on a health and economic basis. Methods: This research evaluated Italian dentists from 11 to 18 April 2020, using a questionnaire submitted via Google Forms (Alphabet, Mountain View, CA, USA). It consisted of different investigations about sociodemographic aspects, profession-related characteristics, knowledge about COVID-19 infection transmission modalities, symptoms, and attitude in treating potentially infected patients. Statistical analysis was performed using the Pearson chi^2^ test and Student t-test. The α-level was fixed at *p* = 0.05. All data were analyzed with STATA 16 (StataCorp LP, College Station, TX, USA). Results: 1500 dentists (664 men and 836 women) completed the questionnaire. The majority of respondents declared having been trained in infection prevention procedures (64.3%) but not specifically to prevent the spread of COVID-19 (48.7%). A total of 57.2% declared that they were not trained sufficiently to restart working after lockdown, with a significantly higher prevalence (Pearson chi^2^ test, *p* < 0.001) among women (62.3%) than men (50.9%). Conclusion: Italian dentists were informed correctly on the mode of transmission but partially missed COVID-19 symptoms. Dentists considered the virus infection highly dangerous, and they were not confident in being able to work safely. The lack of precise operating guidelines creates uncertainties on infection control measures and appropriate personal protective equipment (PPE) use. The participants revealed apprehension for their health and the current and future economic situation of their practices.

## 1. Introduction

Coronavirus disease 2019 (COVID-19) is a pandemic disease caused by severe acute respiratory syndrome coronavirus 2 (SARS-CoV-2) which is spreading worldwide. The epidemic started in December 2019 in the city of Wuhan, China. The World Health Organization (WHO) declared it a pandemic on 11 March 2020 [1]. Italy has been one of the first and most hit European countries since the end of February, and it has had more than 170,000 cases and more than 20,000 deaths [2,3]. The prime minister declared a period of lockdown in accordance with the Italian National Institute of Health, with regional administrations trying to limit the spread of the disease, identify suspected cases, and activate quarantine measures [4,5,6].

Concerning dental practices, dentists were asked to limit their activities to non-deferrable urgent care such as pulpitis, abscess, and broken removable prosthesis [7]. The Centers for Disease Control and Prevention (CDC), the American Dental Association (ADA), and the World Health Organization (WHO) recommended practice guidelines for dental clinicians to control the COVID-19 infection [8,9,10,11]. These recommendations include the use of personal protective equipment (PPE) such as FPP2 and FPP3 for aerosol-producing procedures, single-use protective clothing, protective eyewear, frequent and accurate surface disinfection, ventilation of the operating room, and washing hands with alcoholic solutions. Preliminary mouth washing with chlorhexidine or hydrogen peroxide can significantly reduce bacteria and virus load of the oral cavity; however, the use of dental dams is strongly recommended in restorative and endodontic procedures [5,12]. New protocols are needed to guarantee a safe workplace: telephonic or virtual triage, social distancing between patients, avoiding journals or redundant pieces of furniture in waiting rooms, and reducing the presence of partners, relatives, and parents [13]. At the present date, there are no guidelines regarding how to manage routine dental care at the end of the lockdown period.

The aim of this investigation is two-fold. Firstly, the authors aimed to evaluate dentists’ knowledge regarding COVID-19. Secondly, we investigated dentists’ perception of the risks associated with COVID-19, their attitudes to reopening their activities, and how they judge the institutional intervention on a health and economic basis.

## 2. Materials and Methods 

This research evaluated Italian dentists and orthodontists from private dental offices, National Healthcare Service, and University Clinics from 11 to 18 April 2020. The questionnaire used by the research published by Khader et al. [7] was adapted to the Italian situation and submitted via Google Forms (Alphabet, Mountain View, CA, USA). The Google Forms questionnaire was filled in by 1500 dentists from all the parts of the country. The questionnaire was written in Italian and consisted in different investigations about sociodemographic aspects, profession-related characteristics, knowledge about COVID-19 infection transmission, symptoms, and attitude in treating potentially infected patients (29 items, set up as multiple choice and linear scales). The questionnaires were anonymous, to maintain the privacy and confidentiality of all the information reported in the present research.

### Statistical Analysis

Some of the answers were codified as dichotomous variables, namely as Yes/No responses, or in general as categorical variables, when a multiple-choice selection had been requested. The data were summarized as percentages. The continuous variables corresponding to scores were expressed as mean, standard deviation, and 95% confidence interval. The normal distribution of data was graphically evaluated. The comparison between the groups of female and male dentists was performed by means of the Pearson Chi^2^ test for the categorical variables and Student t-test for independent data for the continuous variables. The α-level was fixed at *p* = 0.05. All data were analyzed with STATA 16 (StataCorp LP, College Station, TX, USA).

## 3. Results

The survey was accessible online from 11 to 18 April 2020. The number of dentists who completed the questionnaire was exactly 1500. The respondents comprised 664 men and 836 women. The age distribution was different between genders (Table 1; Pearson chi^2^ test, *p* < 0.001). Twenty-four percent of the dentists questioned work in the northwest of Italy, 27.2% in the northeast, 27.1% in central Italy, and 21.7% in the southern area and the Islands (Sicily and Sardinia).

Two hundred and forty-three of the respondents (16.2%) work exclusively as orthodontists (67 males, 10.1%; 176 females, 21.0%) while the remaining interviewees practice other dental disciplines in combination, or not, with orthodontics (Table 2). One thousand and thirty-five respondents (69%) had been working for more than 10 years, 223 (14.9%) from 5 to 10 years, and 242 (16.1%) less than 5 years. Sixty-three percent were awarded a postgraduate diploma (63% of men and 64% of women) and 58% have a dental practice (70% men and 48% of women). The majority of respondents declared having been trained in infection prevention procedures (64.3%) but not specifically to prevent the spread of COVID-19 (48.7%), with no difference in percentages between women and men (Pearson chi^2^ test, *p* > 0.005). Moreover, they considered educating laypeople regarding virus spread prevention beneficial (score 9.7 over 10; SD, 0.7).

A particularly large percentage of the sample was not involved in treating COVID-19 patients (68.5%). In fact, 93.9% worked in a private practice, 3.5% at university departments, and only 2.6% in the National Healthcare Service. Dentists expressed the belief that they were reasonably up to date regarding COVID-19 (score 7.1 over 10, DS 1.8) while 59.1% of the sample were informed about appropriate personal protective equipment (PPE) use. As regards an understanding of COVID-19 symptoms, only 13.2% of the respondents correctly selected all the known symptoms while completing the questionnaire. Nevertheless, a high percentage of the sample identified almost all symptoms and were aware of the asymptomatic transmission of COVID-19 (Table 3). The difference in percentage of correct answers between men and women was not relevant. A similar result was obtained for the question regarding groups of people at high risk of infection (Table 3).

The majority of respondents (70.2%) correctly chose all the known ways of transmission (coughing and sneezing, handshaking, and touching surfaces) and an additional 21.5% of interviewees answered only “coughing and sneezing”. All the dentists questioned (99.5%) were informed regarding the unavailability of a COVID-19 vaccine. In Table 4, dentists’ sources of information have been listed. The majority of respondents were trained by doctors and dentists, who had gained expertise in the field. Furthermore, the National Health Institute guidelines were considered as the reference source.

Despite this, 57.2% of respondents declared that they were not trained sufficiently to restart working after lockdown, with a significantly higher prevalence (Pearson chi^2^ test, *p* < 0.001) among women (62.3%) than men (50.9%). In fact, 65.7% of the sample would have declined to treat a patient suffering from a runny nose and cough. In the case of dental emergency only, some respondents would have treated the patients, protecting themselves with proper use of PPE (8.2%), or by prescribing the polymerase chain reaction (PCR) test (8.2%) after the emergency treatment. One hundred and forty-four respondents (9.6%) would have referred the patient to the National Healthcare Service.

Dentists’ attitudes towards COVID-19 have been summarized in Table 5. The virus infection has been defined as dangerous, meaning that dentists were not confident in being able to work safely. Working in a pandemic setting generates concern. At the same time, having the dental office closed is problematic. Income loss is a critical aspect of this situation, where the Government action plan to respond to the pandemic (score 5 over 10, SD 2.3) and financial support (score 3.5 over 10, SD 2.2) were considered insufficient. The stratification of the scores for gender resulted in a slightly higher concern regarding dentists’ personal situations, with a marginally more positive evaluation of the Italian Government’s measures among female dentists than their male counterparts (Student t-test for independent data; *p* < 0.05).

## 4. Discussion

This study used a questionnaire to evaluate Italian dentists’ perceptions and attitudes in the COVID-19 infection from 11 to 18 April 2020, more than a month after the outbreak in Italy. The questionnaire was filled in by 1500 dentists (664 men and 836 women). A total of 24% of the dentists questioned work in the northwest, 27.2% in the northeast, 27.1% in central Italy and 21.7% in the southern area and the Islands. The authors aimed to understand the perceptions and knowledge of Italian dentists on the COVID-19 pandemic one month after the start of the lockdown period.

Italian dentists were informed correctly on the mode of transmission but partially missed COVID-19 symptoms. The lack of precise operating guidelines creates uncertainties on infection control measures and appropriate PPE use. The participants revealed apprehension for their health and the current and future economic situation of their practices.

In our sample, women are more numerous than men, evidencing a sort of selection bias, probably due to the social network dissemination of the questionnaire. However, the age distribution is representative of the current characteristics of Italian dentists with an increasing percentage of women in the recent years and a sample of older men.

More than 90% of the respondents work as private practitioners, evidencing that the National Healthcare System and University Departments provide only a minor part of dental treatments in Italy (6.10%). For this reason, only a minor part of the sample considered themselves involved in treating COVID-19 patients (31.5%). The high prevalence of private practice partially explains the significant apprehension for the economic situation of their activities and personal finance. The income loss was considered a critical aspect of this situation, and the Government action plan was considered inadequate both from community health and economic perspectives.

The majority of respondents declared having been trained in infection prevention procedures (64.3%). The authors expected a higher percentage since infection prevention procedures are essential for every healthcare provider in the dental field. About half of the sample (51.3%) considered themselves specifically prepared to prevent the spread of COVID-19, even if, at the present date, no specific guidelines for dental care providers are available from the National Health Institute.

Moreover, dentists expressed the belief that they were reasonably up-to-date regarding COVID-19, while a significant part of the sample (40.9%) was not informed about appropriate PPE use, and only 13.2% of the respondents had correctly selected all the known symptoms while completing the questionnaire. The high percentage of private practices, the consideration of income loss as a critical aspect of the situation, combined with a partial knowledge of PPE use and symptoms, concern the authors regarding the attention that could be given in the near future to infection control procedures. Dental practices present considerably high liabilities and expenses. A prolonged lockdown may result in different colleagues going out of business. There is also an ethical issue to highlight, on how long practices can postpone the need for dental care of our patients in trying to reduce the virus’ spread.

Dentists considered the virus infection highly dangerous, and they were not confident in being able to work safely. The major part of the sample answered that they would refuse to treat patients presenting with a cough and runny nose. In case of emergency, some dentists would refer patients to the National Healthcare Service or only treat the patient while using appropriate PPE. The authors of the present research highlight that, at the moment, there is no consensus regarding the appropriate PPE, and there is no research for specific treatment for COVID-19 management in dental practice [9]. The current approach to COVID-19 consists in preventing the infection, reducing the risk of transmission, early diagnosis, and quarantine of infected or suspected patients (in particular asymptomatic patients or those presenting mild symptoms) [10]. The participants of this questionnaire correctly reported all these aspects. Finally, dentists considered educating laypeople regarding virus spread prevention beneficial, and that every healthcare provider can help in providing correct information and keeping attention highly focused on social distancing and useful behaviors to reduce the spread of the virus.

## 5. Conclusions

Italian dentists were informed correctly on the mode of transmission but partially missed COVID-19 symptoms. The lack of precise operating guidelines creates uncertainties on infection control measures and appropriate PPE use. The participants revealed apprehension for their health and the current and future economic situation of their practices.

## Figures and Tables

**Table 1:** Age distribution of respondents in the full sample according to gender.

Age	Full Sample	Female Dentists	Male Dentists
below 30 years of age			
*n* (percentage)	181 (12.1%)	120 (14.4%)	61 (9.1%)
30–39 years of age			
*n* (percentage)	414 (27.6%)	269 (32.1%)	145 (21.9%)
40–49 years of age			
*n* (percentage)	417 (27.8%)	243 (29.1%)	174 (26.2%)
above 50 years of age			
*n* (percentage)	488 (32.5%)	204 (24.4%)	284 (42.8%)

**Table 2:** Discipline distribution practiced from respondents in the full sample and in the groups of female and male dentists.

Discipline	Full Sample	Female Dentists	Male Dentists
Orthodontics			
*n* (percentage)	243 (16.2%)	176 (21%)	67 (10.1%)
Restorative Dentistry, Endodontics, Prosthodontics, Oral Surgery			
*n* (percentage)	179 (11.9%)	38 (4.6%)	141 (21.2%)
Restorative Dentistry, Endodontics, Prosthodontics, Oral Surgery, Orthodontics, Pediatric Dentistry			
*n* (percentage)	151 (10.1%)	57 (6.8%)	94 (14.2%)
Restorative Dentistry, Endodontics, Prosthodontics, Oral Surgery, Pediatric Dentistry			
*n* (percentage)	113 (7.5%)	54 (6.5%)	59 (8.9%)
Orthodontics, Pediatric Dentistry			
*n* (percentage)	92 (6.1%)	85 (10.2%)	7 (1.1%)
Restorative Dentistry, Orthodontics, Pediatric Dentistry			
*n* (percentage)	69 (4.6%)	52 (6.2%)	17 (2.6%)
Restorative Dentistry, Endodontics, Prosthodontics			
*n* (percentage)	59 (3.9%)	29 (3.5%)	30 (4.5%)
Restorative Dentistry, Endodontics, Prosthodontics, Pediatric Dentistry			
*n* (percentage)	58 (3.9%)	41 (4.9%)	17 (2.6%)
Restorative Dentistry, Endodontics, Orthodontics, Pediatric Dentistry			
*n* (percentage)	51 (3.4%)	42 (5%)	9 (1.4%)
Restorative Dentistry, Endodontics, Pediatric Dentistry			
*n* (percentage)	51 (3.4%)	42 (5%)	9 (1.4%)
Restorative Dentistry, Endodontics, Prosthodontics, Orthodontics, Pediatric Dentistry			
*n* (percentage)	47 (3.1%)	34 (4.1%)	13 (2%)
Prosthodontics, Oral Surgery			
*n* (percentage)	34 (2.3%)	4 (0.5%)	30 (4.5%)
other single or combined disciplines			
*n* (percentage)	353 (23.5%)	182 (21.8%)	171 (25.7%)

**Table 3:** Percentage of correct answers regarding COVID-19 symptoms and categories of people at high risk of infection in the full sample and in the groups of female and male dentists.

COVID-19 Symptoms	Correct Answers in the Full Sample (Percentage)	Correct Answers among Female (Percentage)	Correct Answers among Male (Percentage)	*p* *
Cough	95%	96%	94%	0.036
Fever	99%	99%	99%	0.485
No symptoms	88%	90%	85%	0.001
Shortness of breath	87%	87%	88%	0.58
Diarrhea	62%	64%	60%	0.146
Red eyes	54%	55%	53%	0.643
Runny nose	41%	41%	40%	0.867
Skin rash	22%	23%	22%	0.581
Joint or muscle pain	75%	74%	75%	0.762
COVID-19 groups at risk				
Elderly people	94%	94%	94%	0.708
Chronic disease patients	90%	90%	89%	0.741
Immunosuppressed patients	92%	92%	91%	0.335
Children	94%	94%	95%	0.866
Healthcare workers	94%	94%	94%	0.758
Men	42%	42%	41%	0.954
Travelers	36%	35%	38%	0.32

* Pearson chi^2^ test.

**Table 4:** Sources of information regarding COVID-19 in the full sample and in the groups of female and male dentists.

Source of Information	Full Sample	Female Dentists	Male Dentists	F vs. M
	Percentage	Percentage	Percentage	*p* *
Expert physicians	70%	70%	70%	0.76
National Health Institute	70%	70%	70%	0.931
Expert dentists	70%	69%	71%	0.46
Radio and television	54%	55%	53%	0.385
Social networks	49%	49%	49%	0.937
Newspapers	49%	49%	48%	0.523
Friends and relatives	8%	8%	8%	0.568
Scientific literature	7%	8%	5%	0.029
Online learning	5%	6%	4%	0.095
Dental associations	3%	3%	2%	0.232
Other	2%	2%	2%	0.876

* Pearson chi^2^ test.

**Table 5:** Dentists’ attitude score (range 1 to 10) toward COVID-19 in the full sample and in the groups of female and male dentists.

Question	Full Sample	Female Dentists	Male Dentists	F vs. M
	Score (range 1–10)	Score (range 1–10)	Score (range 1–10)	(*p* *)
How dangerous is COVID-19?				
Mean (SD)	8.0 (1.5)	8.1 (1.5)	7.8 (1.6)	< 0.001
95% CI ^†^	7.9 to 8.0	8.0 to 8.2	7.7 to 7.9	
How confident are you at treating a suspected case of COVID-19?				
Mean (SD)	2.7 (2.2)	2.5 (2.1)	3.1 (2.4)	< 0.001
95% CI ^†^	2.6 to 2.9	2.3 to 2.6	2.9 to 3.3	
How worried are you about going back to work and consequently being at risk of contagion?				
Mean (SD)	7.3 (2.2)	7.5 (2.2)	7.1 (2.3)	0.002
95% CI ^†^	7.2 to 7.5	7.4 to 7.7	7.0 to 7.3	
How worried are you about the income loss of your dental office because of lockdown?				
Mean (SD)	8.3 (2.0)	8.3 (2.0)	8.2 (2.1)	0.2915
95% CI ^†^	8.2 to 8.4	8.2 to 8.5	8.1 to 8.4	
How worried are you about the consequence of income loss for you and/or your family because of lockdown?				
Mean (SD)	7.9 (1.1)	8.0 (2.0)	7.8 (2.2)	0.0078
95% CI ^†^	7.8 to 8.0	7.9 to 8.2	7.6 to 7.9	
How satisfied are you with Government measures for response to pandemics?				
Mean (SD)	5.0 (2.3)	5.1 (2.3)	4.8 (2.3)	0.0015
95% CI ^†^	4.9 to 5.1	5.0 to 5.3	4.6 to 4.9	
How satisfied are you with Government measures for response to economic crisis?				
Mean (SD)	3.5 (2.2)	3.7 (2.2)	3.3 (2.2)	0.0004
95% CI ^†^	3.4 to 3.6	3.5 to 3.8	3.1 to 3.4	

* Student t-test for independent data, ^†^ 95% CI is 95% Confidence Interval.
